# Supplementary material for: The causal relationship between the human gut microbiota and pyogenic arthritis: a Mendelian randomization study
Source: Front Cell Infect Microbiol. 2024 Nov 26;14:1452480. doi: 10.3389/fcimb.2024.1452480 (PMC11629706; doi:10.3389/fcimb.2024.1452480)
Supplement: Supplementary file 1 [file DataSheet1.zip › mendelian test/ebi-a-GCST90027563.csv_forest.pdf]

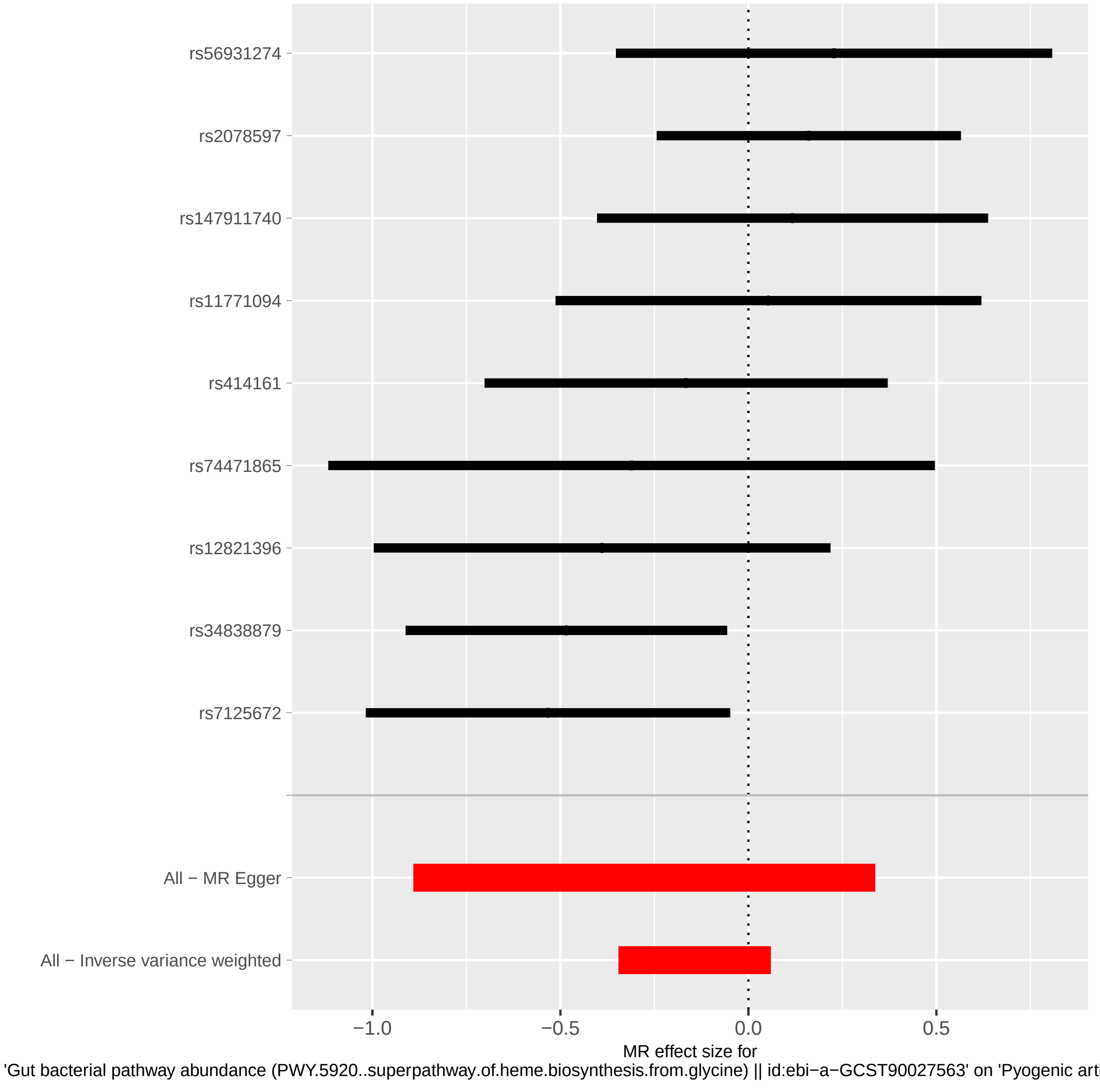
rs56931274
rs2078597
rs147911740
rs11771094
rs414161
rs74471865
rs12821396
rs34838879
rs7125672
All − MR Egger
All − Inverse variance weighted
−1.0
−0.5
0.0
0.5
MR effect size for
'Gut bacterial pathway abundance (PWY.5920..superpathway.of.heme.biosynthesis.from.glycine) || id:ebi−a−GCST90027563' on 'Pyogenic art
